# Echocardiography in left atrial thrombosis

**DOI:** 10.1002/ccr3.7153

**Published:** 2023-03-30

**Authors:** Tara Moghaddasfar, Hamed Vahidi, Maryam Faramarzpour, Farnoosh Larti

**Affiliations:** ^1^ Department of Cardiology, Imam Khomeini Hospital Complex Tehran University of Medical Sciences Tehran Iran

**Keywords:** echocardiography, left atrial thrombosis

## Abstract

Atrial fibrillation is one of the major predisposing factors in developing left atrial thrombosis, leading to morbidity and mortality. Echocardiography plays a paramount role in this condition's detection and subsequent treatment.

## 1 CASE PRESENTATION

Atrial fibrillation is associated with a low‐flow state and increased thrombogenicity in the left atrium (LA). Multiple echocardiographic presentations, including thrombosis, sludge, and smoke formation, have been demonstrated in just one *biplane* transesophageal echocardiographic view, along with normal structures, that is, pectinate muscles and Coumadin ridge, which are highly educational for cardiologists.

**FIGURE 1:**
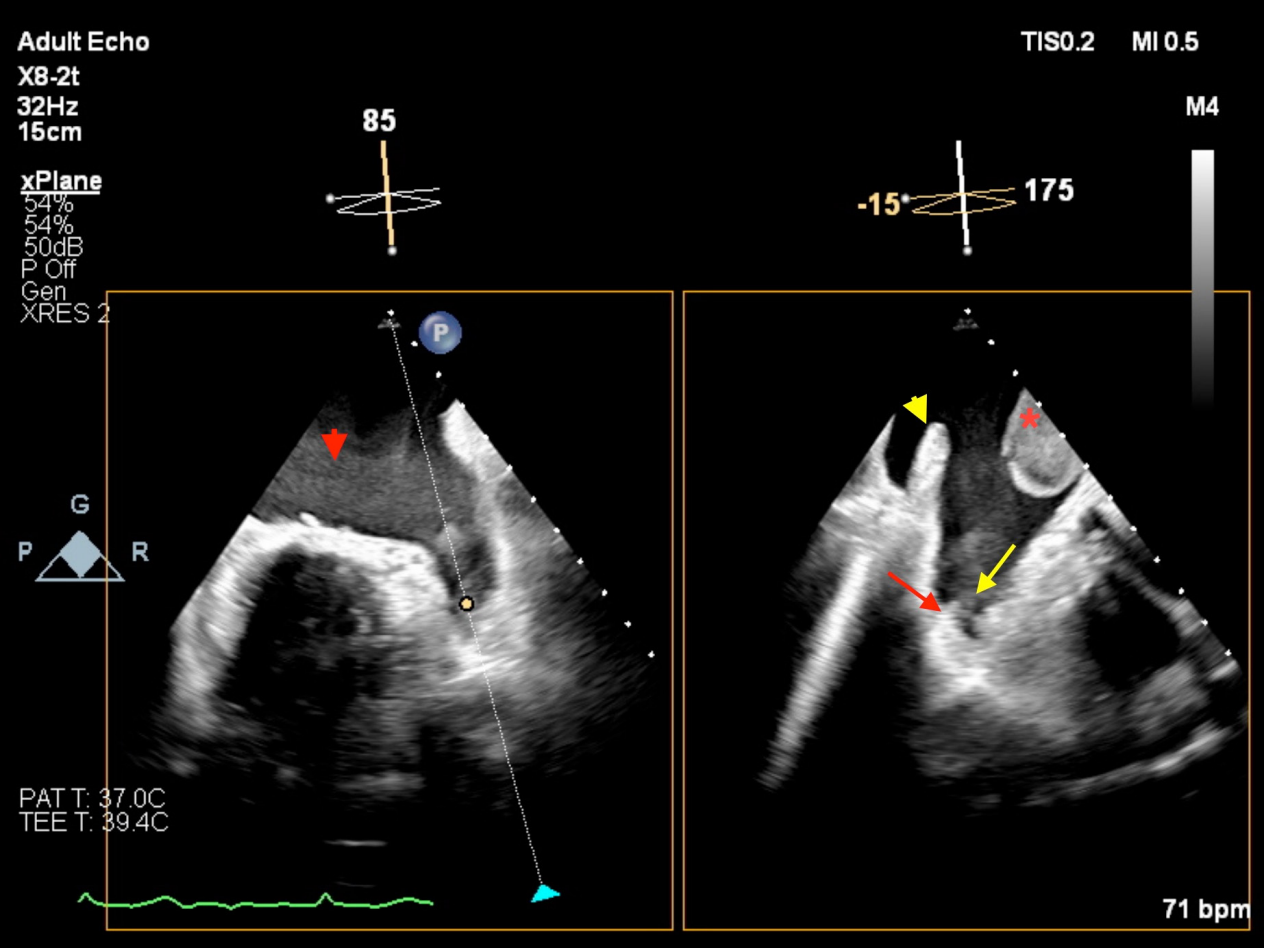
Biplane view in transesophageal echocardiography demonstrating left atrial thrombosis left atrial smoke, left atrial appendage sludge, pectinate muscle in the left atrial appendage, and Coumadin ridge. Red arrow: Pectinate muscle; Yellow arrow: Left atrial appendage sludge; Red arrowhead: Left atrial smoke; Yellow arrowhead: Coumadin ridge; Red star: Left atrial thrombosis.

A 54‐year‐old man with a history of mitral valve repair 9 years ago due to a flail posterior mitral valve leaflet was admitted to our hospital. He also had a history of permanent atrial fibrillation (AF) and was taking warfarin, but his International Normalized Ratio (INR) was subtherapeutic. Transesophageal echocardiography (TEE) for assessment of LA thrombosis was performed. Interestingly, different echocardiographic presentations of the increased thrombogenicity in the left atrium (LA) have been detected in just one biplane view (Figure 1 and Video S1). A large LA thrombosis, LA appendage sludge, severe spontaneous echo contrast (smoke) in the LA, and normal structures, such as pectinate muscles and Coumadin ridge, were detected.

*Spontaneous echo contrast* (*SEC*) *or smoke* is defined as an echogenic, dynamic, swirling blood flow pattern mainly observed as a marker of a low‐flow state in the LA. The presence and the grade of SEC are related to an increased risk of thrombosis formation in the LA. The severity is graded from 0 to 4+, with grade 0 indicating the absence of echogenicity and grade 4+ indicating severe echogenicity and very slow swirling patterns in the LAA, usually with similar density in the LA cavity.^1^

Among patients with AF, the prevalence of spontaneous echo contrast in LA was 8%, and the rate of sludge was 1%–3.4% based on different studies. 2%–12.4% of patients with AF taking vitamin k antagonists (VKA) may have LA or LAA thrombosis, depending on the study population.^2^

Pectinate muscles are normal ridge‐like structures within the LAA wall that may be difficult to distinguish from LAA thrombosis, specifically at the tip of the LAA. Coumadin ridge is a band‐like structure between LAA and the left upper pulmonary vein that may cause a reverberation artifact resembling a thrombosis in the LAA.^3^

## AUTHOR CONTRIBUTIONS

**Tara Moghaddasfar:** Resources; software; writing – original draft. **Hamed Vahidi:** Resources. **Maryam Faramarzpour:** Conceptualization; data curation; resources. **Farnoosh Larti:** Conceptualization; data curation; methodology; project administration; resources; software; supervision.

## FUNDING INFORMATION

No funding was received for this study.

## CONFLICT OF INTEREST STATEMENT

No conflict of interest was present to be disclosed.

## PATIENT CONSENT STATEMENT

Written informed consent was obtained from the patient regarding this research process.

## Supporting information


Video S1:

## Data Availability

The data of this study are available for assessment.
